# Newly identified APN splice isoforms suggest novel splicing mechanisms may underlie circRNA circularization in moth

**DOI:** 10.1002/2211-5463.12689

**Published:** 2019-07-26

**Authors:** Meijing Gao, Yuan Liu, Yun Wang, Xiao Zhang, Sa Dong, Xianjin Liu

**Affiliations:** ^1^ Institute of Food Safety and Nutrition Jiangsu Academy of Agricultural Sciences Nanjing China; ^2^ School of Food and Biological Engineering Jiangsu University Zhenjiang China; ^3^ Horticulture Department Jinling Institute of Technology Nanjing China; ^4^ School of Horticulture and Plant Protection Yangzhou University China

**Keywords:** alternative splicing, aminopeptidases N, circular RNA, splicing mechanism

## Abstract

Circular RNA (circRNA) have long been considered by‐products of splicing errors, but the coordination of RNA transcription and exon circularization events remains poorly understood. Here, we investigated this question using genes encoding aminopeptidases N (APNs), which are receptors of *Bacillus thuringiensis* toxins, in the cotton bollworm, *Helicoverpa armigera*. We cloned and sequenced the cDNA of ten APN genes (*HaAPN1‐10*) located in the same APN gene cluster, and detected 20 and 14 novel splicing isoforms with exon skipping in *HaAPN1* and *HaAPN3*, respectively, whereas no or very few variants were found in the remaining genes. Further study identified 14 and 6 circular RNA (circRNA) in *HaAPN1* and *HaAPN3,* respectively. Neither novel splicing isoforms nor circRNA were detected in *HaAPN2* and *HaAPN5*. Distinct from the conventional GT/AG splicing signal, short co‐directional repeats were involved in the splicing of the linear and circular isoforms of *HaAPN1* and *HaAPN3*. Identification of the splice sites revealed that the linear isoforms may be related in some way to the circularization. Moreover, phylogenetic analysis and detection of circRNA of the APN gene of the diamondback moth, *Plutella xylostella* (*PxAPN3*), showed that circRNA formation is relatively conserved during the lepidopteran evolutionary process. These results contribute to an improved understanding of lepidopteran APNs and this novel class of insect circRNA.

**Abbreviations**
APN: aminopeptidase N
cicrRNA: circular RNA
gDNA: genomic DNA
ORF: open reading frame
qRT‐PCR: quantitative real‐time PCR
RNAi: RNA interference
RNase R: ribonuclease R

Alternative splicing is an essential post‐transcriptional process by which multiple functional RNA or proteins are generated from a single transcript. A large number of gene isoforms can be generated via alternative splicing, the most striking example of which is the *Drosophila Dscam* gene, which encodes a cell adhesion protein that is important for fly neuronal wiring and immune responses. An incredibly large number of Dscam isoforms (38 016) can be generated via mutually exclusive splicing of 4 cassette exon clusters 1. Alternative splicing patterns constantly change under physiological conditions, allowing organisms to respond to changes in the environment by determining the part of the genome that gets expressed 2. Most alternative splicing events can be grouped into one of four categories: alternative 5′ splice sites, alternative 3′ splice sites, cassette exons and retained introns 3.

The mechanisms of alternative splicing have been extensively studied in recent years. The splicing reaction is catalysed by the spliceosome, which is a large, highly dynamic, multicomponent complex 1. The splicing code is extremely intricate, and recent studies on the correlation between circular RNA (circRNA) formation and exon skipping have provided a new perspective from which to investigate alternative splicing 4, 5, 6. CircRNA have long been considered by‐products of splicing errors, and they are rare and likely lack biological functions in organisms 7. With the advent of high‐throughput sequencing of nonpolyadenylated RNA transcripts, many studies have revealed large numbers of circRNA that are endogenous to eukaryotes (e.g. humans, mice, zebrafish, worms, fruit flies, plants), many of which are abundant and stable 8, 9, 10. CircRNA have no free 3′ or 5′ end, so these RNA cannot be detected by molecular techniques that rely on polyadenylated free RNA ends. Two additional key features of circRNA are an out‐of‐order arrangement of exons known as a ‘backsplice’ and RNase R resistance 11. CircRNA can arise from exons (exonic circRNA) or introns, but exonic circRNA are the most widely studied of these RNA. Although widely discussed, the mechanisms underlying exonic circRNA production remain under intense investigation. Two mechanisms have been proposed for mammalian exonic circRNA formation, namely ‘direct backsplicing’ and ‘lariat intermediate formation’. Both mechanisms involve the backsplice being formed by the canonical spliceosome 12. One study has demonstrated that flanking complementary sequences, including both repetitive and nonrepetitive sequences, play important roles in exon circularization 13. However, the exact mechanism underlying spliceosome‐associated circularization is unclear. In addition, the coordination of RNA transcription and exon circularization events remains poorly understood.

Aminopeptidases N (APNs), which are exopeptidases that catalyse the cleavage of neutral amino acids from the N termini of peptides, are M1‐type zinc‐dependent metalloproteases 14. APNs are abundant in the brush border membrane vesicles (BBMVs) of insect midguts and are widely known as receptors of *Bacillus thuringiensis* (Bt) toxins, although their main function is protein digestion 15. The involvement of APN genes in the mode of action of Cry1A and Cry1C toxins was suggested following knockdown by RNA interference (RNAi), which resulted in reduced susceptibility among lepidopteran larvae 16, 17, 18. In *H. armigera*, which is a moth commonly known as the cotton bollworm, corn earworm or Old World (African) bollworm, seven different APN cDNA sequences have been reported 19, but only APN1 has been confirmed to be involved in Bt resistance. Knockdown of APN1 in *H. armigera* larvae and interference in transfected Sf21 cells revealed the interactions of APN1 with Cry1Ac 20. Later evidence showed that a mutation in APN1 is associated with Cry1Ac resistance in *H. armigera* 21.

Given the importance of APN1 in the mode of action of Bt toxins, we sequenced the *HaAPN1* gene in two *H. armigera* strains and detected a number of splice isoforms in the cDNA samples. We then confirmed that this variation was the result of post‐transcriptional modification of the mRNA with novel and regular splice patterns. Analysis of the other nine *HaAPNs* in the same cluster as *HaAPN1* in the *H. armigera* genome showed that this splicing event is common in APN genes. Further analysis revealed that this type of splicing is likely associated with the formation of circRNA and that novel noncanonical splice signals (short co‐directional repeats) guide this process. Moreover, the identification of the circRNA of *P. xylostella* APN gene (*PxAPN3*) revealed that the formation of APN circRNA is relatively conserved during the evolutionary process. These results expand our knowledge regarding lepidopteran APNs and this novel class of insect circRNA.

## Results

### Common truncated transcripts of *HaAPN1* in *Helicoverpa armigera* strains

**Figure 1:**
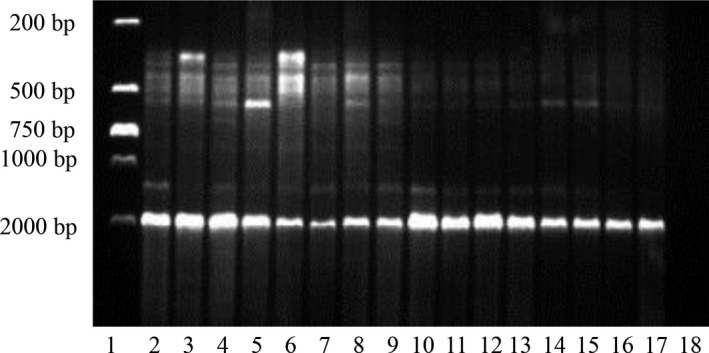
Electrophoresis of nested PCR products of partial cDNA sequences of HaAPN1 from the SCD and An strains of *Helicoverpa armigera*. Lane 1, 2000 bp ladder; lanes 2–9, the cDNA from the SCD strain; lanes 10–17, the cDNA from the An strain; lane 18, the negative control.

**Figure 2:**
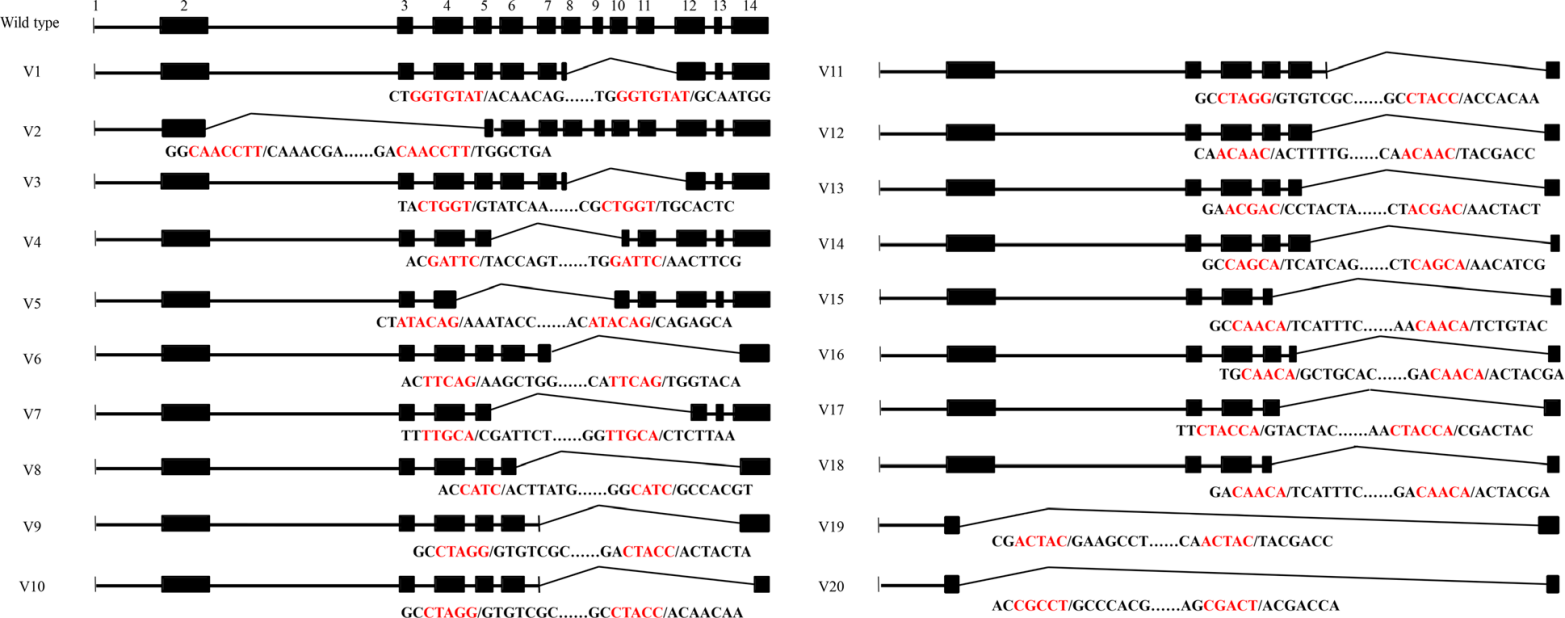
Transcripts of *HaAPN1* in the SCD strain of *Helicoverpa armigera*. Exons are numbered (1–14). Sequences between the black vertical lines (/) are shown for exons missing from transcripts, and red letters denote co‐directional repeats in the splice sites.

**Figure 3:**
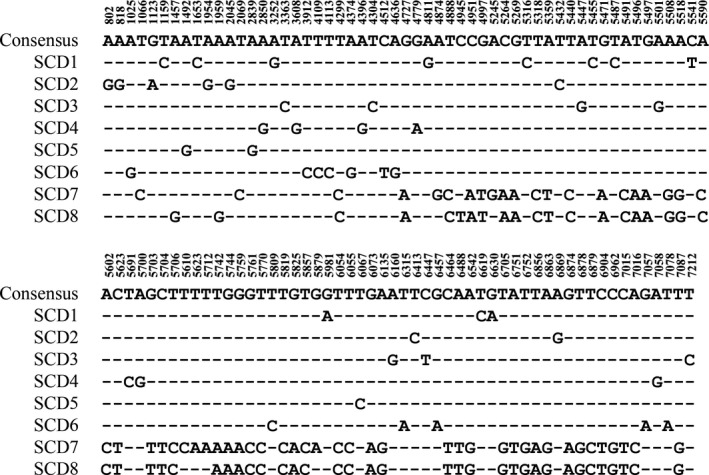
Alignment of partial gDNA sequences of *HaAPN1* from eight larvae of the SCD strain. Dashes indicate that the bases are the same as those of the consensus sequence.

Lepidopteran APNs constitute a protein family that is composed of different classes. In the context of Bt resistance in lepidopteran species, APN1 has received much attention 21, 22. Given the important role of this protein in the resistance of *H. armigera* to Bt, specific primers were designed to amplify the cDNA of *HaAPN1* from the SCD strain, which is a susceptible strain of *H. armigera* that was derived from insects from the Ivory Coast, Africa, over 30 years ago and was maintained in the laboratory without exposure to insecticides or Bt toxins. Extra bands were observed among the nested PCR products obtained with two pairs of primers, namely HaAPN1‐1 and HaAPN1‐2 (Table S1, Fig. 1). The results of sequence analysis indicated that the 20 different sequences obtained from eight larvae of the SCD strain were novel and severely disrupted. These variations resulted in truncation of amino acid residues or ORF frame shifts, leading to premature termination of translation (Fig. 2, Table 1). We then sequenced *HaAPN1* gDNA fragments (corresponding to bp 4‐3042 of the cDNA sequences) amplified from the genomic DNA derived from the eight larvae mentioned above, and no deletions were detected (Fig. 3). The results revealed that the 20 variations likely resulted from RNA splicing rather than mutation of the genomic DNA sequence.

**Table 1:** Linear transcript isoforms of *HaAPN1* from SCD, An and LF256 strains

Isoform	Deletion region	Premature stop codon	Predicted MW (kD)	Number of clones		
				SCD	An	LF256
Wild‐type	–	No	112.8	111	59	45
V1	1637‐2295	Yes	62.5	1	2	0
V2	460‐1125	No	87.1	3	0	1
V3	1633‐2403	No	83.7	7	8	2
V4	1153‐2028	No	80.1	2	2	0
V5	890‐1952	Yes	33.5	6	0	0
V6	1530‐2745	Yes	67.9	2	0	0
V7	1147‐2407	Yes	43.9	1	0	1
V8	1324‐2721	No	61.0	1	2	0
V9	1427‐2883	Yes	58.3	2	0	1
V10	1427‐2904	Yes	57.3	3	0	0
V11	1427‐2937	Yes	56.1	2	0	0
V12	1364‐2912	Yes	55.1	3	0	0
V13	1291‐2846	Yes	54.9	1	0	0
V14	1346‐2982	Yes	51.1	2	1	0
V15	1158‐2831	No	41.0	1	3	0
V16	1221‐2910	Yes	49.9	1	0	0
V17	1058‐2910	Yes	50.7	5	1	0
V18	1058‐2964	Yes	43.2	1	0	0
V19	147‐2852	No	55.1	1	0	0
V20	135‐2934	Yes	8.1	4	2	0

Different variations can originate from a single individual; therefore, we determined the copy number of the *HaAPN1* gene in the SCD strain by a quantitative PCR‐based approach, using genomic DNA as template. The data were normalized to the values for the gene encoding the voltage‐gated sodium channel, two copies of which are present in diploid insect genomes 23. Individual *H. armigera* showed an average relative *APN1* gene copy number of 1.03 (±0.20), indicating that there are approximately 2 copies of the *HaAPN1* gene in the diploid genome of *H. armigera*. This result demonstrated that we could confirm the modification of these variations at the mRNA level.

Moreover, the *HaAPN1* cDNA from other strains, one susceptible strain (An) and one resistant strain (LF256), was sequenced and variations were detected. Eight and four variations were detected in An and LF256 strains, respectively, all of which were also detected in the SCD strain (Table 1, Fig. 1), suggesting that RNA splicing of *HaAPN1* does not occur in only one strain but is a common occurrence among strains; however, isoforms resulting from RNA splicing may differ among individuals. We think this may be resulted by the low frequency of the variants and the limited detection or the random production of these variants.

RNA splicing sites were identified by alignment of the cDNA sequences with the genome sequence. Interestingly, the splice sites were novel, differing from previously reported sites 3. Short co‐directional repeats were consistently present at the splice sites (Fig. 2), which may be associated with post‐transcriptional modification.

### Expression pattern of truncated transcripts of *HaAPN1*

**Figure 4:**
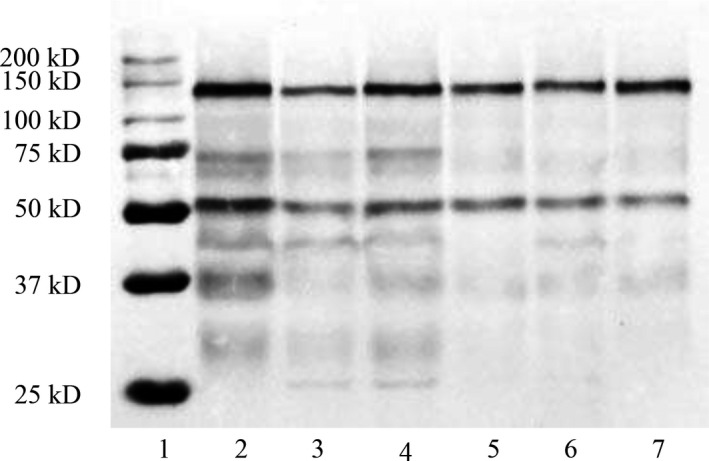
Western blot of HaAPN1. Lane 1, 200 kD ladder; lanes 2–4, BBMVs from the SCD strain; lanes 5–7, BBMVs from the An strain.

Analysis of the nested PCR products revealed that the wild‐type transcript was the most dominant transcript in both strains (Fig. 1), and variant clones accounted for approximately 30% of all the clones that were detected (Table 1). Western blotting was also performed with rabbit polyclonal antibodies that were specific for HaAPN1. The result showed that a band slightly below 150 kD was the main band of the BBMVs, which was consistent with the reported molecular weight (≈ 120 kD) of the HaAPN1 protein 21, and the difference with the predicted size (112.8 kD) may be resulted by the post‐translational modification (Table 1; Fig. 4). The data above suggested that the expression of truncated transcripts did not influence the translation of the wild‐type.

### Analysis of the transcripts of other HaAPN genes in *Helicoverpa armigera*

**Figure 5:**
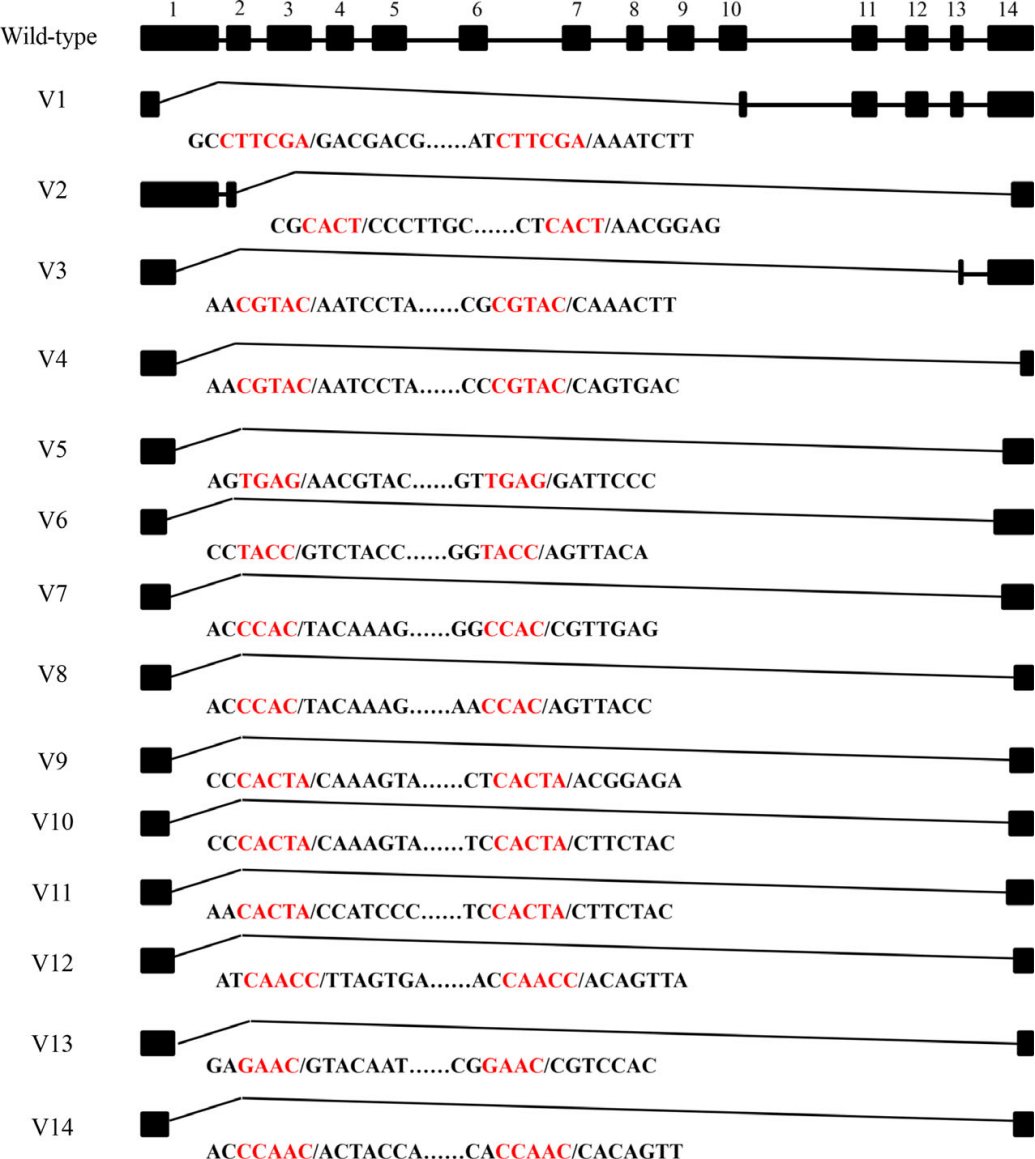
Transcripts of *HaAPN3* in the SCD strain of *Helicoverpa armigera*. Exons are numbered (1–14). Sequences between the black vertical lines (/) are shown for exons missing from transcripts, and red letters denote co‐directional repeats in the splice sites.

**Figure 6:**
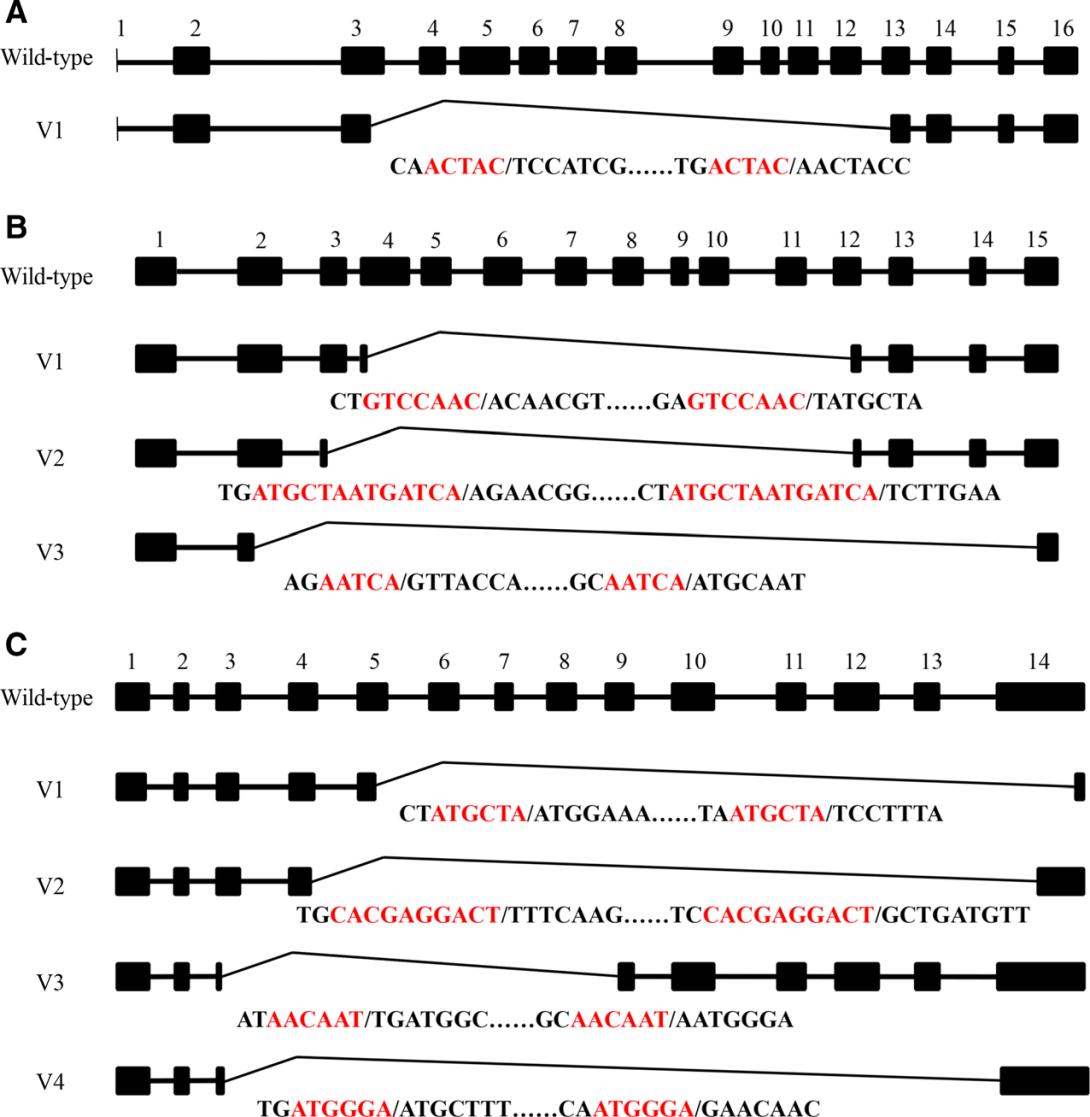
Transcripts of *HaAPN4*,* Ha* APN6 and *Ha* APN9 detected in the SCD strain of *Helicoverpa armigera*. Sequences between the black vertical lines are shown for exons missing from transcripts, and red letters denote co‐directional repeats in the splice site. (A–C) show the transcripts for *APN4*,*APN6* and *APN9*, respectively, in *Helicoverpa armigera*; exons are as numbered above.

We sequenced nine other HaAPN genes located in the same cluster with *HaAPN1*. Fifteen different transcripts, including the wild‐type transcript, were detected for *HaAPN3* (Table 2; Fig. 5), whereas the other genes exhibited no or very few variants. Among these genes, *HaAPN4*,* HaAPN6* and *HaAPN9* had 1, 3 and 4 variants, respectively (Table 3, Fig. 6), and only, the wild‐type transcript isoform was detected for *HaAPN2*,* HaAPN5*,* HaAPN7*,* HaAPN8* and *HaAPN10*. These results suggested that truncated transcripts are common among HaAPNs.

**Table 2:** Linear transcript isoforms of *HaAPN3* from the SCD strain

Isoform	Deletion region (bp)	Premature stop codon	Number of clones
Wild‐type	–	–	55
V1	117‐2301	Yes	1
V2	624‐2851	Yes	2
V3	248‐2713	Yes	5
V4	248‐2963	Yes	1
V5	241‐2827	Yes	3
V6	179‐2758	No	1
V7	208‐2820	No	1
V8	208‐2913	No	3
V9	210‐2852	No	1
V10	210‐2879	No	1
V11	194‐2879	Yes	1
V12	233‐2911	No	1
V13	244‐2939	Yes	1
V14	190‐2910	No	3

**Table 3:** Linear transcript isoforms of *APN4*,* APN6* and *APN9* from the SCD strain

Gene	Isoform	Deletion region (bp)	Premature stop codon	Number of clones
HaAPN4	Wild‐type	–	–	78
HaAPN4	V1	412‐2319	No	2
HaAPN6	Wild‐type	–	–	76
HaAPN6	V1	720‐2408	No	1
HaAPN6	V2	563‐2422	No	1
HaAPN6	V3	345‐2766	Yes	2
HaAPN9	Wild‐type	–	–	74
HaAPN9	V1	732‐2897	No	2
HaAPN9	V2	570‐2649	Yes	1
HaAPN9	V3	306‐1557	Yes	2
HaAPN9	V4	320‐2843	Yes	1

### Identification of circRNA in several HaAPNs

**Figure 7:**
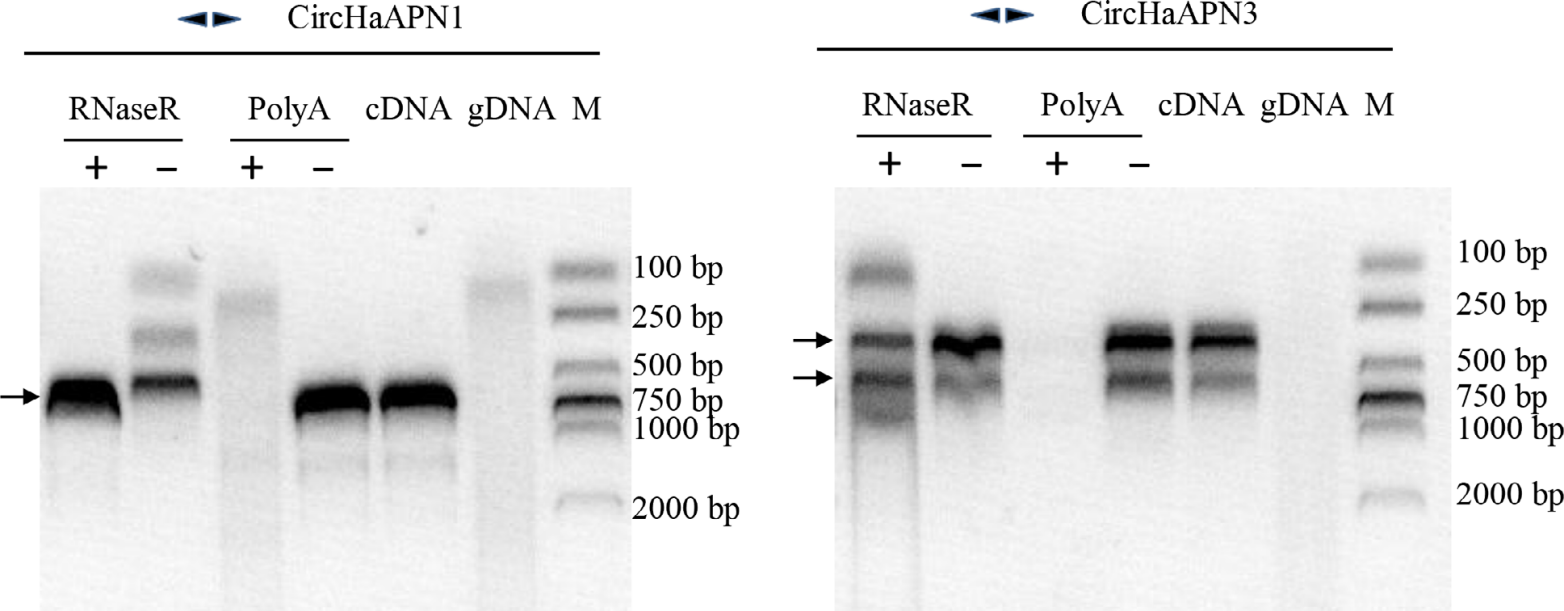
Validation of noncanonical circRNA of *HaAPN1* and *HaAPN3*. Divergent primers were used to amplify from both genomic DNA (as a negative control) and cDNA (regular or with various treatments such as RNaseR digestion and polyA selection). Results were visualized via agarose gel electrophoresis. The arrows mark the target bands.

**Figure 8:**
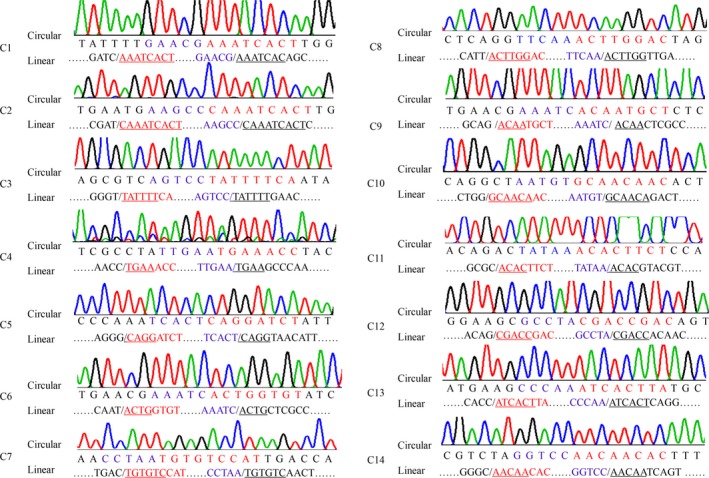
Junctions of circular isoforms and the correlated linear sequences in *HaAPN1*. Corresponding positions in the two kinds of isoforms are indicated by the same colour; the sequences that are underlined are the co‐directional repeats in the splice site.

**Figure 9:**
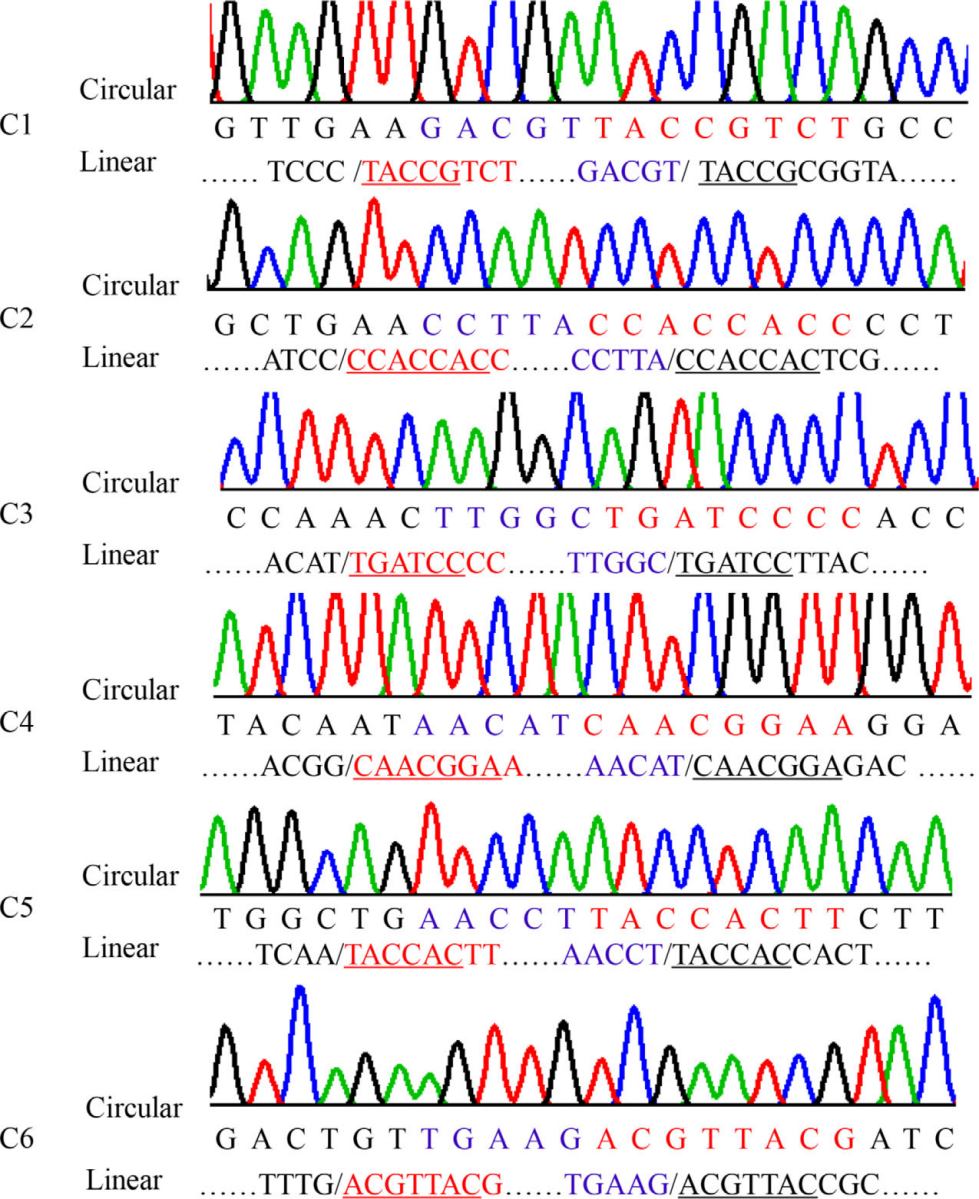
Junctions of circular isoforms and the correlated linear sequences in *HaAPN3*. Corresponding positions in the two kinds of isoforms are indicated by the same colour; the sequences that are underlined are the co‐directional repeats in the splice site.

We identified a number of transcripts for HaAPNs, most of which were truncated, especially those of *HaAPN3*. For this gene, the truncated transcripts lacked most of the exon sequence. We thus attempted to determine the reason underlying the absence of this sequence. As recent studies have reported that exon skipping is correlated with exon circularization 4, we screened the circRNA of four HaAPNs, namely *HaAPN1*,* HaAPN3*,* HaAPN2* and *HaAPN5*. The first two genes exhibited an abundance of truncated transcripts, whereas the other two lacked truncated transcripts. Noncanonical circRNA were validated using divergent primers from both genomic DNA (as a negative control) and cDNA (regular or treated, for example, with RNase R digestion and poly A selection) (Table S2). The reactions were analysed by agarose gel electrophoresis and Sanger sequencing. Head‐to‐tail junctions were detected in the *HaAPN1* and *HaAPN3* genes (Figs 7, 8, 9), whereas no amplification product was obtained for *HaAPN2* and *HaAPN5* with divergent primers (Table S2). For instance, *HaAPN1* and *HaAPN3* produced at least fourteen and six distinct exonic circular RNA, respectively (Figs 8 and 9). We further analysed these circular isoforms and found that multiple exon circularization products could be obtained from single gene loci (Tables 4 and 5); this phenomenon is termed alternative circularization (AC) 13, 24, 25.

**Table 4:** Location of the splicing sites of *HaAPN1* linear and circular isoforms in the gDNA

Isoform	Linear isoforms				Isoform	Circular isoforms			
	Donor		Acceptor			Donor		Acceptor	
	Position (bp)	Exon	Position (bp)	Exon		Position (bp)	Exon	Position (bp)	Exon
V1	5046	exon8	6260	exon12	C1	6693	exon13	5382	exon9
V2	1188	exon2	4150	exon5	C2	6722	exon12	5381	exon9
V3	5042	exon8	6368	exon12	C3	6682	exon13	5187	exon8
V4	4178	exon5	5671	exon10	C4	6715	exon13	5117	exon8
V5	3786	exon4	5595	exon10	C5	6731	exon13	5093	exon8
V6	4860	exon7	6942	exon14	C6	6698	exon13	5035	exon8
V7	4172	exon5	6372	exon12	C7	6640	intron12	4896	exon7
V8	4486	exon6	6918	exon14	C8	6852	exon14	5080	exon8
V9	4589	exon6	7080	exon14	C9	6698	exon13	4811	exon7
V10	4589	exon6	7101	exon14	C10	6526	exon12	4535	exon6
V11	4589	exon6	7134	exon14	C11	6540	exon12	4470	exon6
V12	4543	exon6	7109	exon14	C12	7132	exon14	5050	exon8
V13	4453	exon6	7043	exon14	C13	6725	exon13	4483	exon6
V14	4508	exon6	7179	exon14	C14	6933	exon14	4537	exon6
V15	4183	exon5	7028	exon14					
V16	4383	exon6	7107	exon14					
V17	4083	exon5	7107	exon14					
V18	4083	exon5	7161	exon14					
V19	875	exon2	7049	exon14					
V20	863	exon2	7131	exon14					

**Table 5:** Location of the splicing sites of *HaAPN3* linear and circular isoforms in the gDNA

Isoform	Linear isoforms				Isoform	Circular isoforms			
	Donor		Acceptor			Donor		Acceptor	
	Position (bp)	Exon	Position (bp)	Exon		Position (bp)	Exon	Position (bp)	Exon
V1	117	exon1	4661	exon10	C1	6364	exon13	175	exon1
V2	728	exon2	6830	exon14	C2	6610	exon14	386	exon1
V3	248	exon1	6592	exon14	C3	6601	exon14	380	exon1
V4	248	exon1	6842	exon14	C4	6650	exon14	365	exon1
V5	241	exon1	6706	exon14	C5	6608	exon14	412	exon1
V7	208	exon1	6699	exon14	C6	6360	exon13	766	exon2
V8	208	exon1	6792	exon14					
V9	210	exon1	6731	exon14					
V10	210	exon1	6758	exon14					
V11	194	exon1	6758	exon14					
V12	233	exon1	6790	exon14					
V13	244	exon1	6818	exon14					
V14	190	exon1	6789	exon14					

The splice sites of circular transcripts were determined by sequence alignment. Interestingly, as observed for the truncated linear transcripts, short co‐directional repeats were present at the splice sites (Figs 8 and 9).

### Complementary sequence identification for circRNA of HaAPN1 and HaAPN3

To identify the complementary flanking sequences, 10 nucleotides of upstream and downstream flanking sequences (20 nucleotides total) of each splice site were extracted and classified as sequence 1 (20‐nucleotide flanking sequence upstream of the circRNA) and sequence 2 (20‐nucleotide flanking sequence downstream of the circRNA). We analysed the sequences with the RNAfold web server (http://rna.tbi.univie.ac.at//cgi-bin/RNAWebSuite/RNAfold.cgi) and found that among the 20 circular isoforms, only eight circRNA had some complementary sequences near the splice sites (Table 6). These complementary sequences were short, ranging in size from 4 to 5 nucleotides (Table 6).

**Table 6:** Complementary sequences surrounding splice sites of circRNA

Gene	Isoform	Flanking sequences upstream of the circRNA		Flanking sequences downstream of the circRNA	
		Sequence 1	Complementary sequences	Sequence 2	Complementary sequences
HaAPN1	C1	AACTACGATCAAATCACTTG	TCAAA	ATTTTGAAC*G*AAATCACAGC	TTTGA
	C2	TAACTACGATCAAATCACTT	–	GAATGAAGC*C*CAAATCACTC	–
	C3	GGGAATGGGTTATTTTCAAT	TCAA	GCGTCAGTC*C*TATTTTGAAC	TTGA
	C4	TTTGACAACCTGAAACCTAC	–	GCCTATTGA*A*TGAAGCCCAA	–
	C5	TGGACTAGGGCAGGATCTAT	–	CCAAATCAC*T*CAGGTAACATT	–
	C6	TGAGCGCAATACTGGTGTAT	GAGC	GAACGAAAT*C*ACTGCTCGCC	GCTC
	C7	CATTGTTGACTGTGTCCATT	TTGAC	TAAAACCTA*A*TGTGTCAACT	GTCAA
	C8	CATCCCCATTACTTGGACTA	–	TCAGGTTCA*A*ACTTGGTTGA	–
	C9	GCAACCGCAGACAATGCTCT	–	GAACGAAAT*C*ACAGCTCGCC	–
	C10	CACTTACTGGGCAACAACAC	–	AGGCTAATG*T*GCAACAGACT	–
	C11	CTGTCAGCGCACACTTCTCC	–	CAGACTATA*A*ACACGTACGT	–
	C12	GTATCAACAGCGACCGACAG	–	GAAGCGCCT*A*CGACCACAAC	–
	C13	CTTCTCCACCATCACTTATG	CTTC	TGAAGCCCA*A*ATCACTCAGG	GAAG
	C14	CTTACTGGGCAACAACACTT	–	GTCTAGGTC*C*AACATTCAGT	–
HaAPN3	C1	CGCTTCTCCCTACCGTCTGC	CGTCT	TTGAAGACG*T*TACCGCGGTA	AGACG
	C2	ACATTGATCCCCACCACCCC	–	CTGAACCTT*A*CCACCACTCG	–
	C3	GAAGGAACATTGATCCCCAC	AAGGA	CAAACTTGG*C*TGATCCTTAC	TCCTT
	C4	CAAGGAACGGCAACGGAAGG	–	ACAATAACA*T*CAACGGAGAC	–
	C5	TCAATCTCAATACCACTTCT	–	GGCTGAACC*T*TACCACCACT	–
	C6	GCCAAGTTTGACGTTACGAT	ACGT	ACTGTTGAA*G*ACGTTACCGC	ACGT

### Identification of circRNA in *PxAPN3*

**Figure 10:**
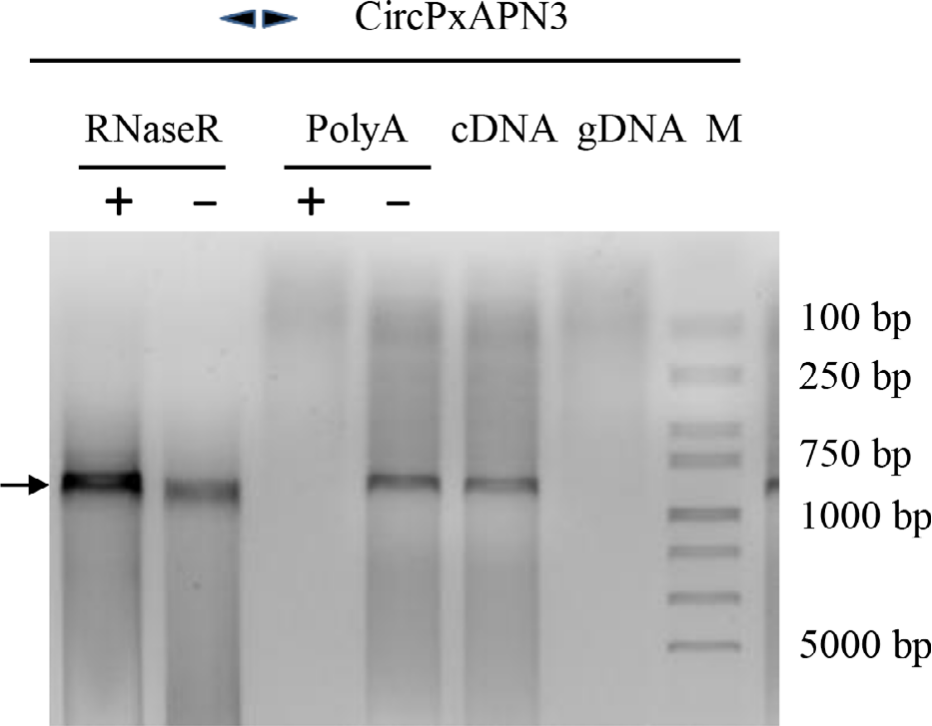
Validation of noncanonical circRNA of *PxAPN3*. Divergent primers were used to amplify from both genomic DNA (as a negative control) and cDNA (regular or with various treatments such as RNaseR digestion and polyA selection). Results were visualized via agarose gel electrophoresis. The arrow marks the target band.

**Figure 11:**
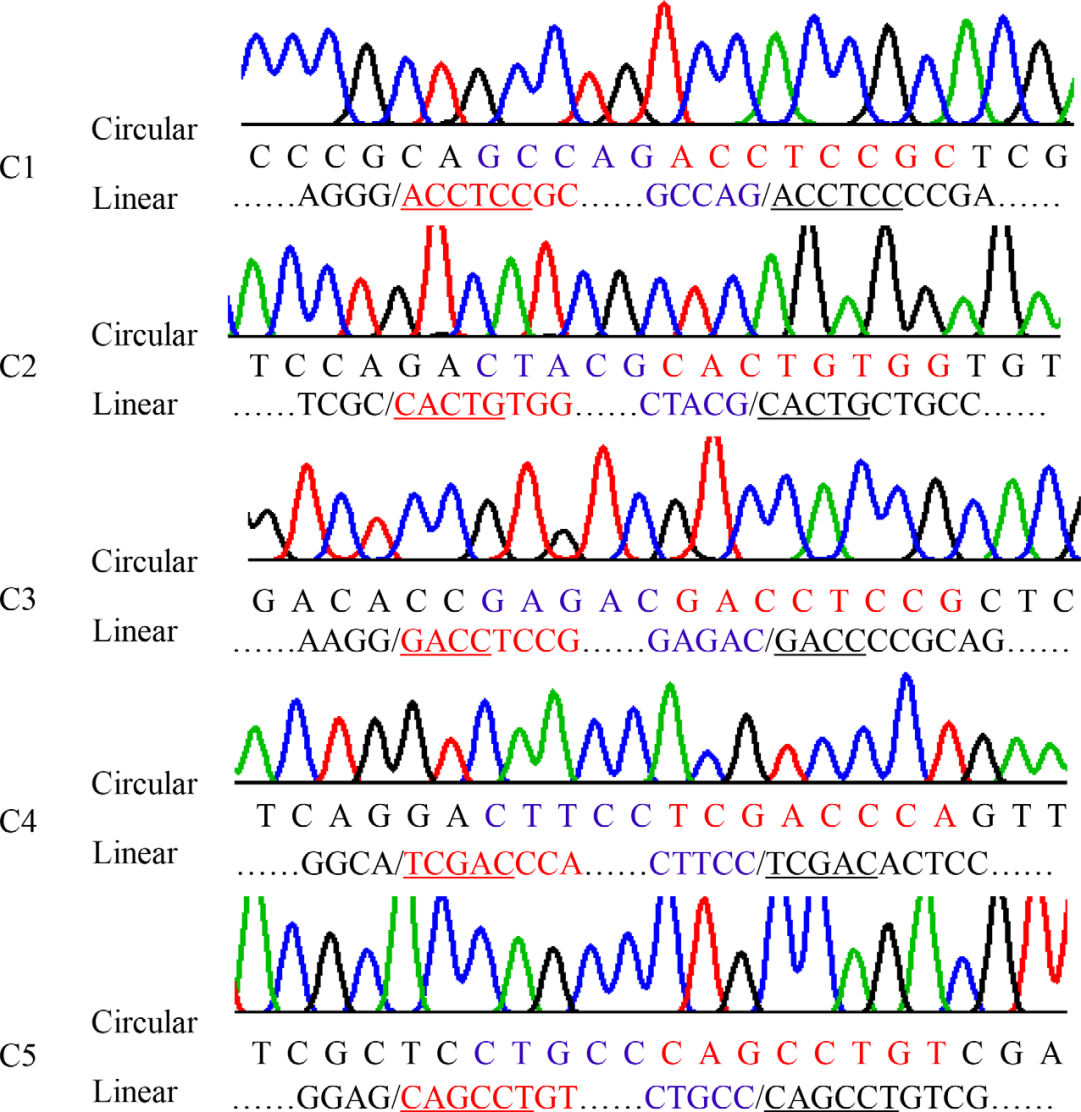
Junctions of circular isoforms and the correlated linear sequences in *PxAPN3*. Corresponding positions in the two kinds of isoforms are indicated by the same colour; the sequences that are underlined are the co‐directional repeats in the splice site.

Several studies have revealed that multiple gene families developed from APN genes before the evolutionary divergence of Lepidoptera 26, 27, 28. Analysis of the phylogenetic tree showed that HaAPN1 and HaAPN3 are more closely related to each other than to other HaAPNs 26, 27, 28, and multiple circular transcripts were detected for these two genes. Therefore, we investigated whether the formation of circRNA derived from HaAPNs was evolutionarily conserved in Lepidoptera. For this investigation, we selected the *PxAPN3* gene because *HaAPN3* is more closely related to *PxAPN3* than to *HaAPN1* 26, 27, 28 and because circular isoforms were also detected for this gene. Five circular isoforms were identified, and similar noncanonical splice signals also guided the formation of circRNA in *PxAPN3* (Figs 10 and 11).

## Discussion

Members of the aminopeptidase N (APN) gene family of the insect order Lepidoptera (moths and butterflies) bind to insecticidal Cry toxins naturally produced by the bacterium *Bacillus thuringiensis*. Various studies on APN1 from *Manduca sexta* 29, *Lymantria dispar* 30, 31, *P. xylostella* 32, *H. armigera* 21, 33 and *Trichoplusia ni* 22 have consistently demonstrated that this protein is one of the midgut receptors for Cry1Ac. During our research on APN1 in *H. armigera*, 21 different linear transcripts were detected from two susceptible strains. Among these transcripts, 20 had novel sequences and were severely disrupted, resulting in truncation of amino acid residues or ORF frame shifts (Fig. 2, Table 1). As an important Bt receptor, modification of HaAPN1 may influence the interaction with Bt toxins 21, 33. In our study, the most common splice variants exhibited premature termination (Fig. 2, Table 1), lacking the domain that binds with Cry1Ac 19. However, these variants showed no preference in susceptible and resistant strains (Table 1) and the wild‐type HaAPN1 remained dominant in the susceptible strains (Figs 1 and 4), and thus, the SCD and An strains had not lost their susceptibility to Bt toxin. Therefore, these truncated transcripts may not influence the interaction with Bt toxins, but the high variability of midgut APN1 in *H. armigera* populations provides genetic potential for the evolution of Bt resistance.

It has been proposed that members of the APN family were derived from gene duplication events that occurred in an ancestral Lepidoptera species 26, 27, 28. We asked whether splicing events also occurred in the other APN genes in *H. armigera*. Therefore, we sequenced nine other APN genes that were located in the same cluster as *HaAPN1*. Fifteen different transcripts, including the wild‐type transcript, were detected for *HaAPN3* (Table 2; Fig. 5), whereas the other HaAPNs exhibited no or very few variants (Table 3; Fig. 6). We then analysed the evolutionary relationships of the ten genes, and the phylogenetic tree showed that HaAPN1 and HaAPN3 diverged more recently than did the other HaAPNs 26, 27, 28, suggesting that the structures of these two genes are very similar. This finding might explain why *HaAPN1* and *HaAPN3* exhibited a large number of variations, while the other genes did not. Furthermore, this finding suggests that the splice mode might be dependent on the structure of the pre‐mRNA.

CircRNA have been extensively studied in recent years and may play specific roles in cellular physiology. Research on the correlation between circRNA formation and exon skipping provides a new perspective from which previously observed splicing events can be explained 4, 5, 6. Among the four HaAPNs screened, *HaAPN1* and *HaAPN3* exhibited different head‐to‐tail junctions (Figs 7, 8, 9), whereas no amplification product was obtained for *HaAPN2* and *HaAPN5* with divergent primers. This finding suggests that circular splicing events may be related to linear splicing events.

The splice sites of the linear truncated transcripts and circular transcripts were identified by sequence alignment. Interestingly, the splice sites were novel, differing from previously reported modes, and short co‐directional repeats were consistently present at the splice sites (Figs 2, 5, 8 and 9). The repeat sequences were not fixed and were at least 4 bp in length. Splice sites for canonical alternative splicing or AC are mostly located in the junctions between exons and introns, and canonical splicing signals, including GT at the upstream end and AG at the downstream end, have been widely assumed to be markers of linear alternative splicing and circRNA 34. In our study, nearly all the splice sites were located in the exon, and non‐GT/AG splicing signals guided the formation of linear and circular splice transcripts (Figs 2, 5, 8 and 9). Co‐existing canonical and noncanonical splice signals have been reported to guide the formation of circRNA 10, 35. However, to date, noncanonical splicing signals have been detected only in plants, and there is little knowledge regarding these signals. The results of our study indicate that further research on this subject is warranted.

Complementary sequences within introns and exons are reported to play important roles at splice sites of circRNA 10, 13. We extracted the sequences flanking the splice sites and found that only 40% (8/20) of the circRNA had some complementary sequences near the splice sites. These complementary sequences were short, ranging in size from 4 to 5 nucleotides (Table 6). Similarly, in *Arabidopsis thaliana* samples, only 37% of the circRNA had some complementary sequences near the splice sites 10. These observations indicate that in addition to complementary sequences, there must exist other elements that guarantee circularization.

**Figure 12:**
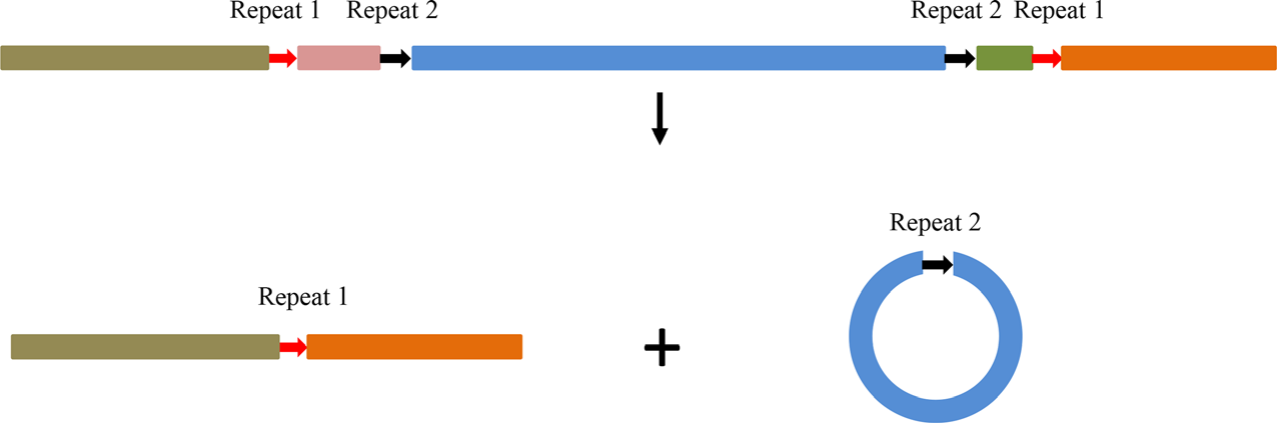
A schematic of the locations of splicing sites in linear and circular isoforms. The red arrows and the black arrows indicate the co‐directional repeats in the linear isoform and circular isoform, respectively, ‘repeat’ means repeat sequence, and the numbers denote different types.

Because of the co‐existence of circular splicing events and linear splicing events, we suspected that the linear variants were by‐products of circular isoform formation. We investigated the specific locations of the splice sites and found that the splice sites of the linear and circular transcripts did not complement each other perfectly. For example, among the circular *HaAPN3* transcripts in sample S, only C4 could be detected, whereas three different truncated linear transcripts were detected in the same sample (V4, V7 and V8). The splice sites of the two types of transcripts did not overlap; in fact, the splice sites of the circular transcripts were located within the linear splice sites (Table 5; Fig. 12). Even though there was no overlap between the splice sites of the linear and circular transcripts, we believe that the linear variants are related to the circular isoforms for two reasons. The first reason is the similarity of the splicing signal: short co‐directional repeats were involved in the formation of both types of transcripts. The second reason is that the splice sites between these two types of transcript were adjacent (Tables 4 and 5). If the circular isoforms were not related to the linear variants, the splice sites of the circular isoforms would have been randomly distributed rather than densely clustered in the splice site‐rich region of the linear variants. For example, for *HaAPN3*, the donor positions of the linear transcripts and the acceptor positions of the circular isoforms were all clustered in exons 1 and 2, and the acceptor positions of the linear transcripts and the donor positions of the circular isoforms were located mainly in exons 13 and 14 (Table 5). We designed different pairs of divergent primers to amplify the *HaAPN3* circular isoforms, and no new splice sites were detected in other exons. Moreover, divergent primers were designed to amplify the circular isoforms of *HaAPN2* and *HaAPN5*, but we did not detect any products. Similarly, based on the data described above, no linear variants were detected for these two genes. For the above‐mentioned reasons, we suspected that the circRNA may be related in some way to linear spliced events.

Several studies have revealed all of the APN genes were derived from gene duplication 26, 27, 28, and we suspected that there existed circRNA corresponding to the ancestral gene. It is possible that during evolution, some APNs lost this circRNA, and some APNs, such as the later ancestor of *HaAPN1* and *HaAPN3*, retained this circRNA. Alternatively, it is possible that circRNA formation did not occur for the ancestral gene and that during the evolutionary process, some APNs, such as the later ancestor of *HaAPN1* and *HaAPN3*, acquired this feature. We know that multiple gene families developed from APN genes before the evolutionary divergence of Lepidoptera, and the amino acid sequence identity of the encoded proteins ranges from 23 to 40% between different clusters of the same species and from 50 to 95% between members of the same cluster in different species 26, 27, 28. Thus, *HaAPN3* shares closer evolutionary relationships with the APN3 of other species than with *HaAPN1*. We selected the *PxAPN3* gene for analysis and detected five circular isoforms with the same noncanonical splice signals (Figs 10 and 11), which proved that there is some conservation for the circRNA formation in the evolutionary process of lepidopteran APNs.

In summary, the results of this study demonstrate that linear and circular splice transcripts are common in several lepidopteran APNs, and the circRNA may be related in some way to linear spliced events. A class of noncanonical splicing signals (short co‐directional repeats) was shown to participate in the formation of the two types of transcripts. Phylogenetic analysis and detection of circular RNA of several APN genes showed that the formation of circular RNA is relatively conserved during the evolutionary process. These results expand our knowledge of lepidopteran APNs and this novel class of insect circRNA. Nonetheless, as the functions of the circular isoforms and truncated linear isoforms and the details of the splicing process remain unknown, further in‐depth studies are needed.

## Materials and methods

### Insects

The SCD strain is a susceptible strain of *H. armigera* that was derived from insects from the Ivory Coast, Africa, over 30 years ago and has been maintained in the laboratory without exposure to insecticides or Bt toxins 36, 37. The An strain, which is another susceptible laboratory strain of *H. armigera*, was derived from a field strain from Anyang, Henan Province, northern China 38. The LF256 strain originated from a field‐captured male, and the resistance to Cry1Ac was autosomal, recessive and 220‐fold relative to susceptible strain SCD 39. Larvae were reared on an artificial diet, and adults were maintained as described previously 36, 37.

Fourth‐instar *P. xylostella* larvae were purchased from Keyun (Henan, China).

### Sequencing of cDNA and DNA samples

For the cDNA samples, total RNA from the midgut tissue of eight fifth instars was individually extracted for each strain using TRIzol reagent following protocols provided by the manufacturer (Invitrogen, Waltham, MA, USA) and reverse transcribed with Moloney murine leukaemia virus reverse transcriptase (Promega, Madison, WI, USA). Specific primers were designed and used to amplify gene fragments (Table S1). PCR products of the expected size were purified using the Wizard DNA purification system (Promega) and cloned into the pGEM‐T Easy vector system (Promega). All clones were sequenced by Invitrogen (Shanghai, China). Reference sequences of the HaAPN genes were downloaded from OGS2 (http://webapollo.bioinformatics.csiro.au/helicoverpa_armigera).

Genomic DNA was isolated from the same fifth instars by phenol–chloroform extraction, and PCR amplification was performed using specific primers (Table S1). Cloning and sequencing were carried out as described above.

### qRT‐PCR assays

To normalize the copy number of the *HaAPN1* gene derived from the SCD strain, genomic DNA was individually extracted as described above. Real‐time PCR samples were prepared in SYBR^®^ Premix Ex Taq^™^ (Tli RNaseH Plus; TaKaRa, Japan), and reactions were carried out using the 7500 RT‐PCR detection system (ABI, Carlsbad, CA, USA). The qRT‐PCR protocol included an initial incubation at 95 °C for 30 s, followed by 40 cycles of amplification at 95 °C for 5 s and 60 °C for 34 s. Data were normalized to the values for the gene encoding the voltage‐gated sodium channel (HaSC), two copies of which are present in diploid insect genomes, using the 2^−ΔΔCT^ method. Primers and PCR conditions were optimized to reduce nonspecific amplification. The PCR efficiency was similar for the target gene (97.2%) and the reference gene (95.7%). The primer sequences are provided in Table S1.

### Midgut BBMV preparation and western blot analysis

Brush border membrane vesicles were prepared from insect midguts by the differential magnesium precipitation method 40. Briefly, for each sample, the midguts of 10 fifth‐instar *H. armigera* larvae were dissected, washed in ice‐cold 0.15 m NaCl solution and then homogenized in 3 mL of MET buffer (pH 7.5, containing 300 mm mannitol, 5 mm EGTA and 17 mm Tris/HCl). Then, 3.5 ml of 24 mm MgCl2 was added, and the samples were incubated on ice for 15 min. The homogenate was centrifuged at 2500 ***g*** for 10 min, and then, the supernatant was transferred into a new tube and centrifuged at 30 000 ***g*** for 30 min. The pellets were collected and dissolved in 800 μL of 10 mm HEPES buffer (pH 7.5, containing 130 mm KCl and 10% glycerol). Prepared BBMVs were kept at −80 °C until used. Protein concentration was determined by the Bradford method with BSA as a standard.

For western blot assays, the BBMV proteins (10 μg) were separated by SDS/PAGE (10%) and transferred to polyvinylidene difluoride (PVDF) membranes (Bio‐Rad, Hercules, CA, USA). The membranes were blocked with 2.5% (w/v) BSA in TBST (pH 7.5, containing 25 mm Tris, 3 mm KCl, 135 mm NaCl and 0.1% Tween‐20) for 1.5 h and then washed three times with TBST. After blocking, all membrane incubations and washes were performed in TBST. Proteins were detected with a polyclonal anti‐HaAPN1 antibody (1/5000; 1 h) and a goat anti‐rabbit secondary antibody coupled with horseradish peroxidase (1/10 000; 1 h) (Abgent, San Diego, CA, USA) followed by the Super Signal chemiluminescent substrate (Thermo, Waltham, MA, USA) according to the manufacturer's instructions. The polyclonal anti‐HaAPN1 antibody was provided by the Institute of Plant Protection, Chinese Academy of Agricultural Sciences (CAAS), China. The antibody specificity was assessed by the expressed HaAPN1 in *Escherichia coli* line (Fig. S1).

### Detection of the circRNA of APN genes

As described above, total RNA from eight *H. armigera* individuals was extracted. One of the eight samples was named Sample S, and the remaining seven samples were combined and named Sample M. Total RNA from ten *P. xylostella* individuals was extracted following the methods described above, and the ten samples were combined for use.

rRNA was removed using the Ribo‐Zero Magnetic kit (Epicentre, Madison, WI, USA). Linear RNA was removed by RNase R (Epicentre) treatment (20 U for 1 h at 40 °C). Linear RNA was extracted with the PolyATtract^®^ mRNA Isolation System III (Promega). Reverse transcription was also performed with the M‐MLV system but with random primers (Promega); primers were designed to amplify the circRNA sequences of the *APN* genes (Table S2). Cloning and sequencing were carried out as described above. Reference sequences of the HaAPN genes were downloaded from OGS2 (http://webapollo.bioinformatics.csiro.au/helicoverpa_armigera). The *PxAPN3* sequence was obtained from NCBI, and the accession number is XM_011556806.

## Conflict of interest

The authors declare no conflict of interest.

## Author contributions

Meijing Gao designed the experiments; Meijing Gao, Yuan Liu, Yun Wang and Sa Dong conducted the experiments; and all authors analysed the data. Meijing Gao and Xianjin Liu wrote the manuscript.

## Supporting information


**Table S1.** Nucleotide primers used to obtain cDNA and gDNA fragments of HaAPNs and to perform qPCR.
**Table S2.** Nucleotide primers used to obtain the circRNA fragments of HaAPNs and PxAPN3.
**Fig. S1.** Western blotting analysis of expressed HaAPNl in *Escherichia coli*. Lane 1, 200 kD ladder; lane 2, epressed HaAPNl in *E. coli*.
