# Supplementary material for: Newly identified APN splice isoforms suggest novel splicing mechanisms may underlie circRNA circularization in moth
Source: FEBS Open Bio. 2019 Jul 26;9(9):1521–35. doi: 10.1002/2211-5463.12689 (PMC6722899; doi:10.1002/2211-5463.12689)
Supplement: Supplementary file 1 — Table S1. Nucleotide primers used to obtain cDNA and gDNA fragments of HaAPNs and to perform qPCR. Table S2. Nucleotide primers used to obtain the circRNA fragments of HaAPNs and PxAPN3. Fig. S1. Western blotting analysis of expressed HaAPNl in Escherichia coli. Lane 1, 200 kD ladder; lane 2, epressed HaAPNl in E. coli. [file FEB4-9-1521-s001.doc]

**Supporting information**

**Table S1.** Nucleotide primers used to obtain cDNA and gDNA fragments of HaAPNs and to perform qPCR.

| Fragment name | Forward sequence (5’-3’) | Reverse sequence(5’-3’) |
| --- | --- | --- |
| For amplifying HaAPNs cDNA fragments | | |
| APN1-1 | ATGGCGAACCGCTGGTACACCCTC | AGCCATATTAACAACGAGAGTC |
| APN1-2 | ATCCAGTGAATACCAACAACTT | AGCCATATTAACAACGAGAGTC |
| APN2 | ACCACCAATCAAATTGTTCT | GCTGAAACTAAGGGTGAATAA |
| APN3 | ATGGCGGCGATAAAACTC | ataaggtgcaaaatagcaccc |
| APN4 | ATGGGTGCCAACATGGTG | AGCCATGCTTCTACAAATGTTC |
| APN5 | ATGCAATTCATCACCATCATAC | GGACAGTACTCGCAATGTTGTTA |
| APN6 | AGGCATTGTATAAAGGACAT | CCACAATAAATACAACAACG |
| APN7 | ATGTTTCGCGCGTTTTTAATTT | AATTAATTATCTTTCTTTGAGA |
| APN8 | ATGTATCAACTGGTGCTGGT | AGCTGTACTGATATTTACTGAGTAT |
| APN9 | ATGGTTCGAAGTAGAATCAAGT | ACACTAGAAGTGCCGTAAAG |
| APN10 | CAAGCCCACGAGCCACTAAA | TTAGGGCGGCAGACAGGGCA |
|  |  |  |
| For amplifying HaAPNs gDNA fragments | | |
| APN1-g1 | GCGAACCGCTGGTACACCCTC | CATTGGGGTTGGTGAGGGCGTG |
| APN1-g2 | ATCCAGTGAATACCAACAACTT | AGCCATATTAACAACGAGAGTC |
|  |  |  |
| For performing qRT-PCR | | |
| HaSC-qPCR | ACCTCACCGATGAGAACTGG | GACAAGAAAGCCCAACCAAA |
| APN1-qPCR | TTGGAAGTCATGGAGCGCTA | TTCCAAACCACATGTGAGCG |

**Table S2.** Nucleotide primers used to obtain the circRNAs fragments of HaAPNs and PxAPN3

| Fragment name | Forward sequence (5’-3’) | Reverse sequence (5’-3’) | Amplification product |
| --- | --- | --- | --- |
| HaAPN1-diver | TCACATGGCTGCAGGCTAAT | GCTTGAGTGATGAGACCCCA | Yes |
| HaAPN3-diver-1 | ACCGCAAGACTACACCACAG | TAGCCTAAGGGGCGTCTGAT | Yes |
| HaAPN3-diver-2 | GTACGAACGCGCATTGAACA | TGTCGAAACGACGCTGGTAA | No |
| HaAPN3-diver-3 | CCTCTATCCTACATCGCGGC | GTAGCAAGGGAGAGCGTAGC | Yes |
| HaAPN2-diver-1 | GTTCTCACAACCACCGCCAG | AATGAAGTCAGTCTTTGCAG | No |
| HaAPN2-diver-2 | GTTCTCACAACCACCGCCAG | GGCATGAAGAGGTACAGTGG | No |
| HaAPN5-diver-1 | CATACCATCTTGGTGTCAGT | CCAGGAGATATCTCTGGAAG | No |
| HaAPN5-diver-2 | CATACCATCTTGGTGTCAGT | CTTCTTTGAGCAGATCAGTC | No |
| PxAPN3-diver | GGCTCGGAGTGGAGGAATGT | CTCGTCGTAGCAAGGGAAGG | Yes |


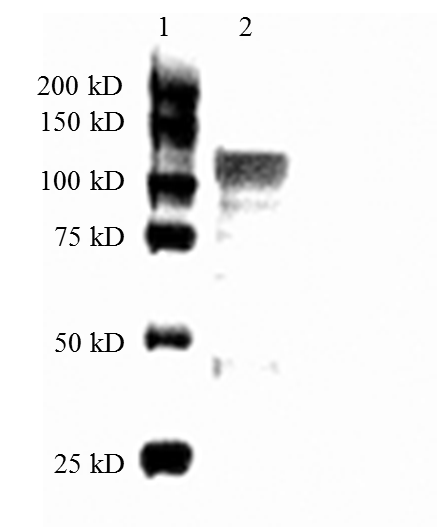


**Fig. S1.** Western blotting analysis of expressed HaAPNl in *Escherichia coli*

Lane 1, 200 kD ladder; lane 2, epressed HaAPNl in *E. coli*
